# A Case of Infective Endocarditis and Pulmonary Septic Emboli Caused by* Lactococcus lactis*

**DOI:** 10.1155/2016/1024054

**Published:** 2016-09-27

**Authors:** Bshara Mansour, Adib Habib, Nazih Asli, Yuval Geffen, Dan Miron, Nael Elias

**Affiliations:** ^1^Pediatric Department, St. Vincent French Hospital, Nazareth, Israel; ^2^Bruce Rappaport School of Medicine, Technion-Israel Institute of Technology, Haifa, Israel; ^3^Pediatric Cardiology Unit, St. Vincent French Hospital, Nazareth, Israel; ^4^Clinical Microbiology Laboratory, Rambam Health Care Campus, Haifa, Israel; ^5^Pediatric Infectious Disease Unit, Haemek Medical Center, Afula, Israel

## Abstract

Infective endocarditis is a rare condition in children with normal hearts. We present here a case of previously healthy eleven-year-old girl with infective endocarditis and pulmonary septic emboli caused by a very rare bacterial etiology (*Lactococcus lactis*). Identification of this pathogen was only made by polymerase chain reaction.

## 1. Introduction

Infective endocarditis (IE) is a relatively rare condition in children but it causes significant morbidity and mortality. Repaired and unrepaired congenital heart disease are associated with a high lifetime risk of infective endocarditis; patients with ventricular septal defect have the highest risk [1]. Acute tricuspid valve endocarditis is rare and usually associated with habitual intravenous self-administration of drugs and more often is associated with central line infections [2].

Septic pulmonary embolism is an uncommon condition in children. Numerous pulmonary infarcts resulting from small emboli may be associated with right-sided bacterial endocarditis, septic thrombophlebitis, and osteomyelitis [3]. Moreover, coexistence of both infective endocarditis and septic emboli is very rare. We present here a child with both IE and septic emboli due to a very rare etiology.

## 2. Case Report

A previously healthy 11-year-old girl presented to the pediatric emergency department with a one-week history of fever, headache, left flank pain, chills, and central cyanosis. On physical examination she was well-looking and afebrile, heart rate was 130 beats/min, blood pressure was 112/70 mmHg, air room saturation was 95–97%, and body weight was 41 kg (50th percentile). Her physical examination was normal. Laboratory analysis showed microcytic anemia with hemoglobin 9.4 g/dL, a white cell count 10.1 × 10^3^/*μ*L, and platelets 211 × 10^3^/*μ*L. C-reactive protein was 15 mg/dL (0–0.5); erythrocyte sedimentation rate was 60 mm/hr. Liver and kidney function tests were normal; creatine phosphokinase was 66 U/L. Urine analysis revealed slight leukocyturia of 25 cells/*μ*L; single blood culture was negative. Chest X-ray showed infiltrate in left lower lobe; X-ray of the sinuses was normal. Oral cefuroxime was prescribed for suspected urinary tract infection and suspected left-side pneumonia, and she was discharged home. Urine culture result was sterile.

**Figure 1:**
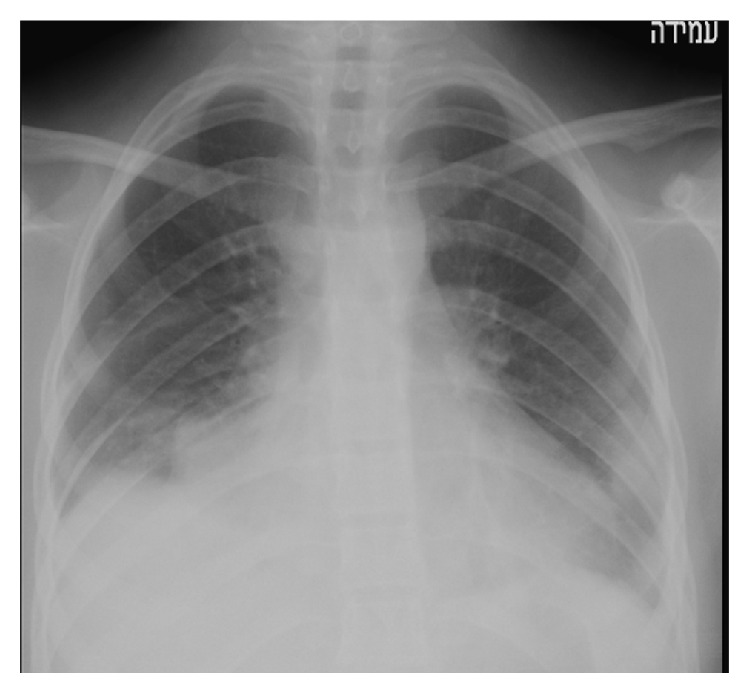
Enlarged perihilar lymph nodes and bilateral lower lobe consolidation.

Two weeks later she presented again to the pediatric emergency department due to a one-day weakness, dyspnea, and pallor without fever. Her physical examination revealed decreased air entry to both lungs and a new 2/6 systolic murmur. C-reactive protein was elevated (20.5 mg/dL) and erythrocyte sedimentation rate was 115 mm/hr. Hemoglobin was 9.4 g/dL, white cell count was 12.9 k/*μ*L, neutrophils were 85%, and elevated lactic dehydrogenase was 1815 IU/L. Chest X-ray showed enlarged perihilar lymph nodes and bilateral lower lobe consolidation, which was interpreted as bilateral pneumonia with a mild bilateral pleural effusion (Figure 1). Urine and blood cultures were taken and she was admitted and treated with intravenous cefuroxime.

**Figure 2:**
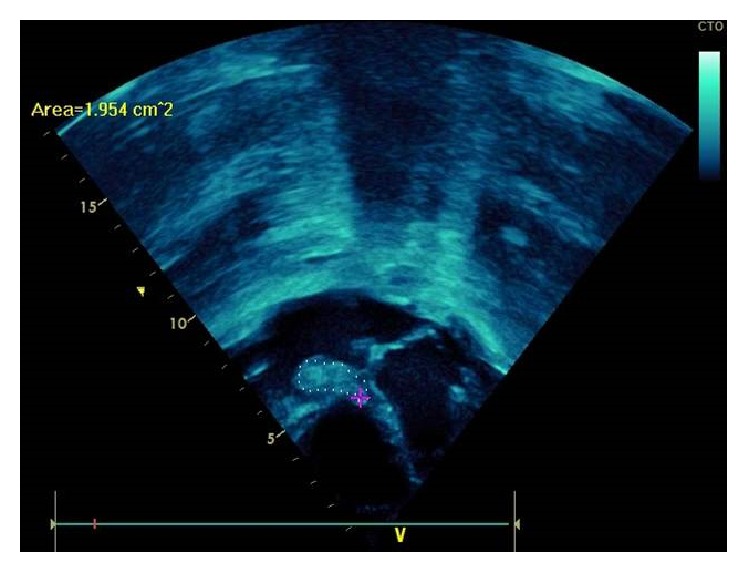
Tricuspid septal leaflet vegetation.

**Figure 3:**
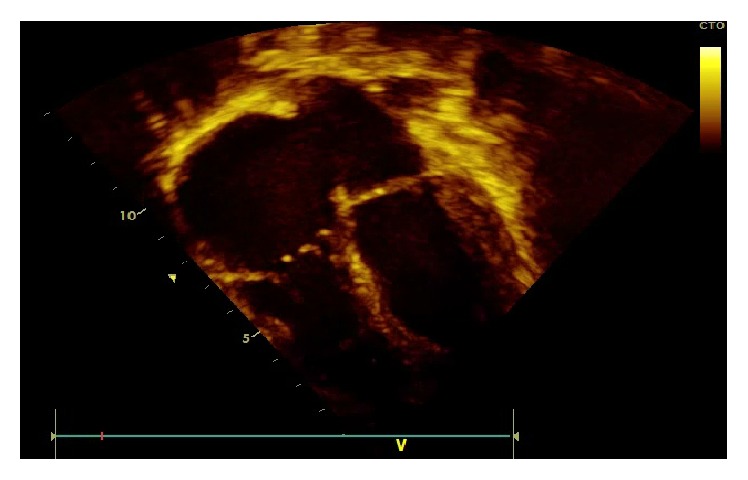
Tricuspid valve vegetation on septal tricuspid valve leaflet.

**Figure 4:**
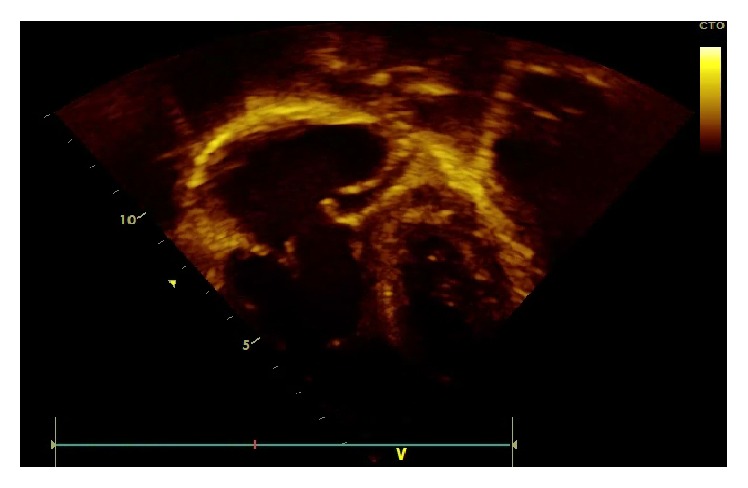
Floating tricuspid valve leaflet and damaged (ruptured) tricuspid valve chordae.

Two days after her admission transthoracic echocardiography (TTE) was done and revealed two vegetations (2.3 × 1 cm and 0.8 × 0.5 cm) attached to the tricuspid valve with a moderate tricuspid regurgitation without other valvular abnormalities (Figures 2, 3, and 4). No ventricular septal defect or patent foramen ovale was demonstrated and intravenous ceftriaxone, gentamicin, and vancomycin were started. Ophthalmologic examination was normal and no other immunologic signs of infective endocarditis were noticed. Other investigations included peripheral blood smear; PCR for respiratory viruses from nasopharyngeal secretion; PCR for Q-fever; serology and blood culture for Brucella, C3 and C4; ANA; and urine Legionella antigen. All these tests were negative and five blood cultures were sterile. We thought that TTE was enough diagnostic and clear so transesophageal echocardiography (TEE) was not performed.

On the fifth day of hospitalization, antibiotic treatment was switched to Ceftriaxone and Daptomycin due to renal function impairment as a result of vancomycin treatment. (We thought at that point that the patient had developed interstitial nephritis due to Vancomycin treatment.) She was discharged on the 22nd day of hospitalization, with significant clinical, laboratory, and radiological improvement. Echocardiography performed prior to discharge revealed a decrease in the size of tricuspid vegetation; however, a small muscular ventricular septal defect became more evident at this time. She ultimately completed a four-week course of intravenous antibiotics.

**Figure 5:**
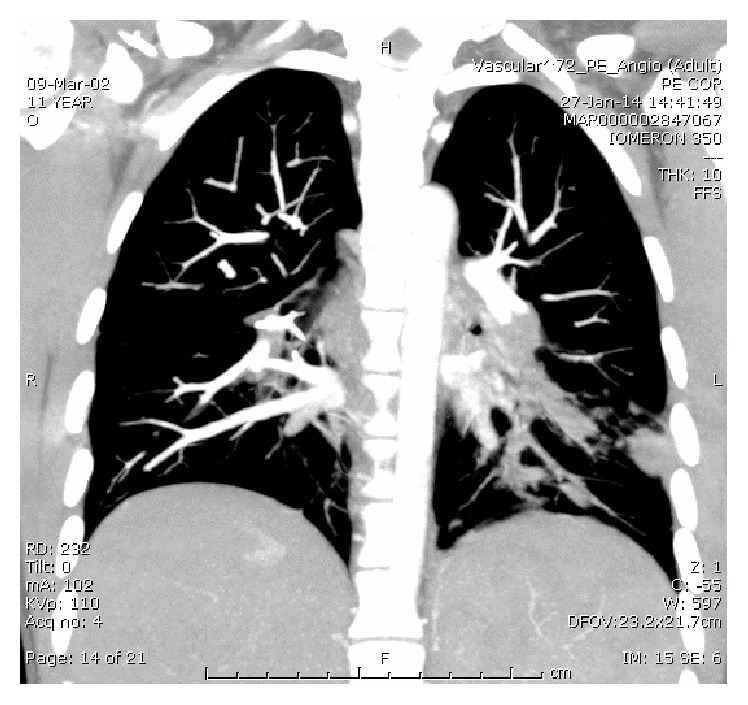
Septic emboli in the bilateral lower pulmonary segments.

Twenty-five days later she was readmitted due to a two-day history of cough, runny nose, effort-induced dyspnea, and left-sided pleuritic chest pain without fever. Vital signs were normal. Physical examination revealed a holosystolic 3/6 murmur. Laboratory analysis revealed CRP 16 and ESR 67. Blood cultures were repeated and she was treated with intravenous cefazolin and ceftriaxone. Chest X-ray revealed middle and left lobe consolidation. Echocardiography revealed a further decrease in vegetation size. Lung CAT scan revealed bilateral consolidation that was diagnosed eventually as septic emboli originating from the tricuspid vegetations, clearly demonstrated by Angio-CT (Figure 5; septic pulmonary emboli originating from the tricuspid vegetation were diagnosed). Echo Doppler of iliac veins, inferior vena cava, and lower extremities veins was normal.

Three blood cultures were again negative (blood cultures at our institute are routinely cultured for 7 days). In spite of that, a broad range pan bacterial PCR test of the 16S rDNA gene performed on the blood culture sample yielded a positive signal [4]. The PCR product was separated by electrophoresis and was then sequenced and analyzed using the Basic Local Alignment Search Tool. The amplicon sequence gave 100% identity to* Lactococcus lactis*. In order to make sure of no contamination during the PCR analysis, we used several controls: a positive control of a known bacterial DNA, a negative control for the DNA extraction process, and a negative control (no template control) for the PCR reaction. All controls were as expected.

Since we had only a positive PCR, but no positive culture, there is no way to perform an antibiogram.

She was discharged on day 15 with the recommendation to complete two weeks intravenous antibiotic treatment with Cefazolin and Ceftriaxone. Additional blood culture sample taken four weeks later tested negative by the same PCR method. She was operated later for tricuspid valve repair without complications and she was followed by a pediatric cardiologist and a cardiac surgery specialist and now she enjoys a good health.

## 3. Discussion

Herein, we report the case of a young girl who was initially reported to be healthy with no congenital heart disease who developed tricuspid infective endocarditis with septic emboli due to a rare etiology,* L. lactis*.

Our patient was diagnosed with infective endocarditis based on modified Duke Criteria [5]. The presented girl had one major criterion, which was evidence of endocarditis on echocardiography (tricuspid vegetation and tricuspid regurgitation) along with three minor criteria: (1) predisposing condition (ventricular septal defect), (2) fever, and (3) pulmonary emboli.

Infective endocarditis in children with congestive heart disease can potentially lead to major complications in and outside the heart. Congestive heart failure occurs in up to 40% of cases and is the leading cause of hemodynamic compromise; this could be due to many factors including the destruction of valves, myocarditis, or arrhythmias [6]. Extracardiac complications are also frequent in up to 43% of cases and are caused by either embolic events or immune phenomena [7]. Vegetation on the tricuspid valve has a high risk of resulting in septic pulmonary emboli, causing various pulmonary complications such as pneumonia and pulmonary abscess [8]. Our patient had developed septic pulmonary emboli originating from the tricuspid vegetations most probably related to the ventricular septal defect (VSD) with left to right blood flow direction.

Right-sided infective endocarditis is associated with congenital heart diseases, drug-users, or central lines infection. History of neither dental treatment, invasive procedures, intravenous drugs, nor central lines was reported in our patient. Initially, our patient was reported as a healthy girl with no previous cardiac malformations; however, as the vegetation got smaller, a hidden small muscular ventricular septal defect (VSD) became evident on echocardiography. The VSD was not infected by vegetations.

Due to our estimation the timing of septic embolism development was during her first admission as the tricuspid vegetation became smaller by echocardiography follow-up.

Of course, rheumatic fever was considered in our differential diagnosis, especially since our patient responded to Jones criteria: one major (carditis) and two minors (fever and elevated ESR and PCR). On the other hand the right-sided cardiac involvement was atypical for this entity.

Repeated blood cultures taken at her presentation and during the course of hospitalization did not grow any pathogen. According to literature, the rate of culture-negative endocarditis varies with different studies, ranging from 2.5% to 31% [5]. In our patient we were able to identify* L. lactis* in blood culture using molecular methods.* L. lactis* did not grow in blood culture using sufficient amount of blood, which could be attributed to previous antibiotic treatment. There was neither history of exposure to unpasteurized milk products nor history of previous gastrointestinal symptoms. Four weeks after treatment, PCR test was negative for bacteria.

*L. lactis* is a mesophilic and microaerophilic fermenting microorganism widely used for the production of fermented food products. It is also occasionally isolated from oropharynx, intestines, or vagina and may even be a part of the normal flora. For a long time it was considered as nonvirulent with low pathogenicity in humans. Early* L. lactis* endocarditis was described recently in a 75-year-old man who had previously undergone mitral valve repair for severe mitral valve prolapse; a literature search shows other isolated cases of* L. lactis*-related endocarditis [9]. In another case report, intravascular catheter-related bacteremia caused by* L. lactis* was described in an infant and treated with Cefotaxime and Vancomycin for 14 days [10].

A recent case report described a sudden death of a four-month-old male infant without congenital heart disease. It was elucidated by postmortem examination that the dead had suffered severe IE, which led him to death. In the microbiological genetic analysis using histological section, the pathogen causing inflammation was identified as* Lactococcus lactis *subspecies [11].

## 4. Conclusions

Congenital heart disease with left to right shunt (like VSD) should be taken into consideration in case of right-sided endocarditis in native-valve, nondrug user patient.

Blood PCR analysis should be performed in case of partially treated or culture-negative endocarditis and also CAT scan in order to exclude pulmonary emboli especially in absence of clinical improvement or unexplained dyspnea despite an appropriate treatment.
